# The Effects of ACTH and Dexamethasone on the Transcriptomic Profile of the Rat Adrenal Gland: An In Vivo Study

**DOI:** 10.3390/cimb48020135

**Published:** 2026-01-27

**Authors:** Emilia Cicha, Małgorzata Blatkiewicz, Karol Jopek, Marta Szyszka, Piotr W. Malendowicz, Anna Olechnowicz, Ludwik K. Malendowicz, Marcin Rucinski

**Affiliations:** 1 Animal Facility, Poznan University of Medical Sciences, Rokietnicka 8, 60-806 Poznan, Poland; emiliacicha@ump.edu.pl; 2 Department of Histology and Embryology, Poznan University of Medical Sciences, 60-781 Poznan, Poland; mblatkiewicz@ump.edu.pl (M.B.); karoljopek@ump.edu.pl (K.J.); mszyszka@ump.edu.pl (M.S.); malendowiczp@gmail.com (P.W.M.); aolechnowicz@ump.edu.pl (A.O.); marcinruc@ump.edu.pl (M.R.); 3 Department of Bioinformatics and Computational Biology, Poznan University of Medical Sciences, Swiecickiego 6, 60-781 Poznan, Poland

**Keywords:** adrenal cortex, ACTH, glucocorticoids, transcriptome, microarray, steroidogenesis, Nr4a, immediate-early genes, mitochondria, SREBP

## Abstract

The hypothalamic–pituitary–adrenal (HPA) axis plays a pivotal role in regulating stress responses through ACTH-stimulated glucocorticoid production. The transcriptional programmes underlying temporal adaptation to prolonged ACTH exposure and glucocorticoid feedback remain incompletely characterized. Adult male Wistar rats were subjected to acute ACTH stimulation (single injection, 1 h) to elicit an immediate transcriptional response, prolonged ACTH exposure (three injections over 36 h) as a repeated exposure, or Dexamethasone treatment (three injections over 36 h). Plasma corticosterone levels were subsequently measured using an enzyme-linked immunosorbent assay (ELISA). The adrenal transcriptome profiling was performed using Affymetrix arrays. Differentially expressed genes (DEGs; |fold change| ≥ 1.8, adjusted *p* < 0.05) were analyzed using limma, followed by pathway and network analyses. Acute ACTH exposure resulted in the induction of 569 DEGs (357 upregulated), including immediate-early genes (Nr4a family, AP-1 factors), cAMP-PKA-CREB signalling components, and heat shock proteins. Prolonged ACTH resulted in 98 DEGs (predominantly downregulated), including the suppression of mitochondrial genes and upregulation of Polycomb repressive complex 2 components, suggesting epigenetic transcriptional attenuation. Dexamethasone treatment yielded 75 DEGs with selective suppression of SREBP-mediated cholesterol biosynthesis and uptake pathways. Twelve genes were downregulated by both prolonged ACTH and Dexamethasone, including sterol metabolism and interferon-stimulated genes. Acute and prolonged ACTH exposure engage distinct transcriptional programmes. Acute stimulation activates immediate-early genes and stress responses, while prolonged exposure suppresses mitochondrial gene expression through transcriptional dampening mechanisms. Dexamethasone is associated with the inhibition of cholesterol metabolism via SREBP pathway suppression. These findings illuminate HPA axis adaptation and glucocorticoid-induced adrenal suppression.

## 1. Introduction

The hypothalamic–pituitary–adrenal (HPA) axis is a neuroendocrine system that coordinates the organism’s response to stress. Hypothalamic corticotropin-releasing hormone (CRH) has been shown to stimulate the release of adrenocorticotropic hormone (ACTH) from the anterior pituitary, which in turn acts on the adrenal cortex to promote glucocorticoid synthesis and secretion [1,2]. In rodents, corticosterone is the primary glucocorticoid, whereas cortisol predominates in humans. The distribution of glucocorticoids within the body involves a regulatory loop that maintains hormonal homeostasis. This regulatory loop involves negative feedback at the hypothalamic and pituitary levels [3].

ACTH exerts its effects on the adrenal cortex through binding to the melanocortin 2 receptor (MC2R), a G protein-coupled receptor that activates adenylyl cyclase and increases intracellular cyclic AMP (cAMP) concentrations [4,5]. The resulting activation of protein kinase A (PKA) leads to the phosphorylation of multiple downstream targets, including the transcription factor cAMP response element-binding protein (CREB). Phosphorylated CREB has been observed to bind to cAMP response elements (CREs) in the promoters of target genes, thereby initiating transcriptional programmes that support steroidogenesis [6]. While these signalling pathways are well-defined at the molecular level, their integration into coordinated, genome-wide transcriptional responses in vivo, particularly under sustained ACTH stimulation, remains incompletely understood. In addition to the canonical cAMP-PKA-CREB pathway, ACTH signalling engages mitogen-activated protein kinase (MAPK) cascades and calcium-dependent pathways that contribute to the full spectrum of adrenocortical responses [7].

The process of glucocorticoid biosynthesis in the adrenal cortex is contingent upon the coordinated expression and activity of steroidogenic enzymes localized in both the mitochondria and the endoplasmic reticulum. Cholesterol, the obligate precursor for all steroid hormones, is delivered to the inner mitochondrial membrane by steroidogenic acute regulatory protein (StAR), where cytochrome P450 side-chain cleavage enzyme (CYP11A1) catalyzes its conversion to pregnenolone [8]. Subsequent enzymatic steps involving 3β-hydroxysteroid dehydrogenase (HSD3B), 21-hydroxylase (CYP21A2), and 11β-hydroxylase (CYP11B1) generate cortisol or corticosterone depending on the species [9]. It is important to note that steroidogenic capacity is influenced by more than just enzymatic activity. Transcription regulation of cholesterol availability and mitochondrial function play a significant role as well. Therefore, alterations in gene expression represent a critical layer of adrenal responsiveness. The adrenal cortex obtains cholesterol through two distinct mechanisms: de novo synthesis via the sterol regulatory element-binding protein (SREBP) pathway and uptake from circulating lipoproteins through low-density lipoprotein receptor (LDLR) and scavenger receptor class B type 1 (SR-BI) [10].

The adrenal response to ACTH manifests in distinct temporal phases. Acute ACTH stimulation has been demonstrated to induce rapid steroidogenic output within minutes through mobilization of cholesterol stores and activation of existing enzymatic machinery [11]. This immediate response is accompanied by the induction of immediate-early genes, including members of the Nr4a family of orphan nuclear receptors and AP-1 transcription factors, which orchestrate subsequent transcriptional changes [12,13]. Prolonged or repeated exposure to ACTH has been shown to elicit additional adaptive responses, including adrenal hypertrophy and altered sensitivity to further stimulation [14]. Despite the extensive characterization of acute ACTH-driven steroidogenic signalling, the comprehensive transcriptional programmes underlying the transition from acute to prolonged ACTH stimulation in vivo remain incompletely defined, particularly with respect to distinct temporal patterns of ACTH exposure.

Exogenous glucocorticoids have been demonstrated to suppress the HPA axis through negative feedback mechanisms operating at multiple levels. In the hypothalamus and pituitary, glucocorticoids have been shown to inhibit CRH and ACTH secretion, respectively, thereby reducing adrenal stimulation [15]. Direct effects on the adrenal cortex have also been described, although the molecular mechanisms underlying these effects are less well understood. Prolonged use of glucocorticoid therapy has been demonstrated to result in adrenal atrophy and the suppression of endogenous cortisol production. These effects may persist for extended periods following the discontinuation of treatment [16]. Although glucocorticoid-induced adrenal suppression is well-recognized clinically, the transcriptional programmes mediating these effects at the adrenal level have not been systematically characterized using genome-wide approaches in vivo, limiting mechanistic insight into therapeutic side effects and recovery dynamics.

Previous transcriptomic studies have examined adrenocortical responses to ACTH in cultured cell lines and isolated adrenal cells. Schimmer and colleagues identified over 1000 transcripts regulated by ACTH in Y1 mouse adrenocortical tumour cells, demonstrating that PKA-dependent signalling accounts for approximately 56% of ACTH-regulated genes [17]. Studies in primary human adrenocortical cells have characterized ACTH-induced gene expression changes, identifying upregulation of steroidogenic enzymes, transcription factors, and signalling molecules [18]. In the rat, microarray analyses have revealed zone-specific transcriptome profiles of the adrenal gland, including differential expression patterns between parenchymal and interstitial cells [19]. Further studies have characterized transcriptomic changes in response to gonadectomy and sex steroid replacement [20], compensatory adrenal growth following unilateral adrenalectomy [21], and the effects of culture conditions on the transcriptomic and proliferative response of primary rat adrenocortical cells to ACTH [22]. However, direct in vivo comparisons of transcriptional responses to distinct temporal patterns of ACTH exposure, as well as parallel assessment of glucocorticoid-mediated suppression, remain scarce.

Accordingly, the aim of the present study was to characterize the transcriptional responses of the rat adrenal cortex to acute ACTH stimulation (1 h after a single injection) for immediate transcriptional response, prolonged ACTH exposure (12 h after three injections at 0, 12, and 24 h) as repeated exposure, and Dexamethasone treatment to assess transcriptional programmes associated with glucocorticoid-mediated suppression. The objective of this study was to utilize genome-wide microarray analysis to identify the distinct molecular signatures associated with each condition and to elucidate the transcription factor networks and biological pathways that mediate these responses. It was hypothesized that acute and prolonged ACTH exposure would engage qualitatively different transcriptional programmes, and that glucocorticoid treatment would produce a unique pattern of gene regulation reflecting negative feedback mechanisms at the adrenal level.

## 2. Materials and Methods

### 2.1. Animals and Experimental Design

Adult male Wistar rats with a body weight ranging from 160 to 200 g were obtained from the Laboratory Animals Breeding Centre, Department of Toxicology, Poznan University of Medical Sciences. The animals were housed under identical standard laboratory conditions with a 12:12 h light-dark cycle at 22–24 °C, with ad libitum access to standard chow and tap water. Prior to the commencement of the experiment, the animals were habituated to human contact and handled with the utmost care to mitigate stress. All animal experiments were conducted in accordance with institutional guidelines and approved by the Local Ethics Committee for Animal Experimentation (approval number: 27/2025; date of approval: 25 April 2025).

The animals were randomly assigned to four experimental groups (n = 3 per group, 12 animals in total). The number of animals used in the study was consistent with widely accepted standards for transcriptomic analyses and was minimized to the lowest level necessary to achieve sufficient statistical power, while enabling optimal use of the collected biological material. The control group (C) received subcutaneous injections of physiological saline solution at 0, 12, and 24 h and were decapitated 12 h after the last injection. The acute ACTH group (ACTH A) was administered a single bolus subcutaneous injection of ACTH (Synacthen, Novartis, Basel, Switzerland; 5 μg/rat) and were decapitated 1 h post-injection. The prolonged ACTH group (ACTH P) received subcutaneous injections of ACTH (5 μg/rat) at 0, 12, and 24 h and were decapitated 12 h after the last injection. The Dexamethasone group (DEX) received subcutaneous injections of Dexamethasone (Dexaven, Pharmaceutical Company Jelfa SA, Jelenia Góra, Poland; 5 μg/rat) at 0, 12, and 24 h and were decapitated 12 h after the last injection. The designated time points were selected to encompass discrete regulatory phases. A 1-h ACTH exposure was employed to assess immediate early transcriptional responses while limiting secondary effects. A 12-h interval following the final Dexamethasone dose was designed to evaluate sustained glucocorticoid-driven transcriptional alterations and early recovery processes. These choices were guided by prior studies on ACTH signalling dynamics and glucocorticoid feedback in the adrenal cortex. The ACTH dose of 5 μg/rat was selected on the basis of its extensive utilization in rodent studies examining acute ACTH responses and its documented ability to robustly activate adrenal steroidogenesis and immediate early gene expression without inducing supraphysiological overstimulation. A Dexamethasone dose of 5 μg/rat was selected to ensure effective suppression of the HPA axis via glucocorticoid negative feedback while minimizing excessive pharmacological effects, as evidenced by consistent inhibition of endogenous corticosterone production in rodent models. The use of equivalent doses for ACTH and Dexamethasone further enabled the direct comparison of their opposing regulatory effects on adrenal function. All animals were decapitated between 11:00 a.m. and 12:00 p.m., and adrenal glands were immediately excised and processed for RNA extraction within comparable time frames across both experimental and control groups. Animals were euthanized by rapid decapitation without prior anesthesia, a method selected to avoid anesthesia-induced activation or suppression of the HPA axis and associated confounding effects on glucocorticoid secretion and stress-responsive gene expression. The rapid decapitation, as performed by personnel with the requisite experience and training, employs a technique that results in immediate loss of consciousness, thereby minimizing pain and distress while preserving the neuroendocrine state required for reliable assessment of ACTH- and Dexamethasone-dependent transcriptional responses. The animals were gently handled, and tissue collection was completed within minutes to limit procedure-induced stress and prevent secondary alterations in stress-responsive gene expression. Trunk blood was collected into EDTA-coated tubes for the purpose of determining plasma corticosterone levels. 

### 2.2. Plasma Corticosterone Measurement

Plasma samples were stored at −80 °C until analysis. The quantification of corticosterone concentrations was performed using a commercially available enzyme-linked immunosorbent assay (ELISA) kit (Enzo Life Sciences, Cat. No. ADI-900-097, Farmingdale, NY, USA). This process was conducted in accordance with the manufacturer’s instructions. A standard curve ranging from 15 to 2250 ng/mL was established by plotting optical density (OD) against log-transformed concentration values. Absorbance readings were subsequently performed at 450 nm using a microplate reader. The determination of sample concentrations was achieved through the interpolation of OD values onto the standard curve using a four-parameter logistic (4PL) regression model implemented with the drc package (version 3.0-1) in R [23]. All samples were analyzed in duplicate, and results are expressed as ng/mL. Statistical differences among groups were evaluated using the Kruskal–Wallis test.

### 2.3. RNA Isolation and Quality Control

Total RNA was extracted from adrenal glands using TRI Reagent (Sigma-Aldrich, St. Louis, MO, USA) according to the manufacturer’s protocol, followed by purification using NucleoSpin Total RNA Isolation columns (Qiagen GmbH, Hilden, Germany). The quantity of RNA was determined by measuring optical density at 260 nm using a NanoDrop spectrophotometer (Thermo Scientific, Waltham, MA, USA). The purity of RNA was assessed by the 260/280 nm absorbance ratio, with only samples showing ratios ≥ 1.8 accepted for further analysis. RNA integrity was verified using a Bioanalyzer 2100 (Agilent Technologies, Inc., Santa Clara, CA, USA), with RNA integrity numbers (RINs) ranging from 8.5 to 10 (mean: 9.2). RNA samples were diluted to a final concentration of 100 ng/μL for microarray experiments.

### 2.4. Microarray Expression Analysis

Gene expression profiling was performed using the Affymetrix Rat Gene 1.1 ST Array platform. Total RNA (100 ng) from each sample underwent two rounds of sense cDNA amplification, biotin labelling, and fragmentation using the GeneChip WT Plus Reagent Kit (Affymetrix, Santa Clara, CA, USA) following the manufacturer’s instructions. Biotin-labelled cDNA fragments (5.5 μg) were hybridized to Affymetrix Rat Gene 1.1 ST Array Strips at 45 °C for 20 h. Subsequent to hybridization, the array strips underwent a washing and staining procedure utilizing the Fluidics Station of a Gene Atlas System (Affymetrix). Thereafter, scanning was conducted using the Imaging Station of the Gene Atlas System. Preliminary quality control analysis was performed using Affymetrix Gene Atlas Operating Software. CEL files passing quality control criteria were exported for downstream bioinformatics analysis.

### 2.5. Microarray Data Processing and Statistical Analysis

The data assumptions were addressed through the implementation of statistical methods appropriate for transcriptomic data, in accordance with standard quality control and normalization procedures. All bioinformatics analyses were performed using the R programming language (version 4.5.1) [24] with Bioconductor packages [25]. The raw CEL files were imported and processed using the oligo packages [26]. Gene expression values were calculated using the Robust Multiarray Average (RMA) normalization algorithm [27] implemented in the affy library [28], which performs background correction, quantile normalization, and probe summarization. Biological annotation was obtained from the ragene11sttranscriptcluster.db Bioconductor package and merged with normalized expression data. For differential expression analysis, genes were filtered based on interquartile range (IQR) variance (cutoff = 0.35) using the genefilter package [29] to remove low-variance transcripts. Additionally, genes with a median expression < 4 and variance < 0.05 were excluded from further analysis. Linear modelling and statistical testing were performed using the limma package [30]. A design matrix was constructed to model the four experimental groups, and contrasts were defined to compare each treatment group against the control group (ACTH A vs. Control, ACTH P vs. Control, and DEX vs. Control). Empirical Bayes moderation was applied to calculate moderated t-statistics and log of differential expression. *p*-values were adjusted for multiple testing using the Benjamini–Hochberg false discovery rate (FDR) method [31]. Differentially expressed genes (DEGs) were identified using the following criteria: |fold change| ≥ 1.8 and adjusted *p*-value < 0.05. Principal component analysis (PCA) was performed on the top 1000 most variable genes using the prcomp function with centering and scaling. Visualization was performed using the factoextra [32] and ggbiplot [33] packages.

### 2.6. Gene Ontology and Pathway Analysis with DAVID

Gene Ontology (GO) enrichment analysis was performed using the Database for Annotation, Visualization, and Integrated Discovery (DAVID) v6.8 [34,35]. Rat Entrez Gene IDs corresponding to DEGs were converted to human orthologs using the homologene package in R [36]. The mapping success rates were acute ACTH (535/569 genes mapped, 94%), prolonged ACTH (92/98 genes mapped, 94%), and Dexamethasone (71/75 genes mapped, 95%). Genes failing to map were excluded from subsequent pathway and network analyses using human-annotated databases. Separate analyses were conducted for upregulated and downregulated genes in each comparison. Enrichment analysis included GO terms for Biological Process (BP FAT), Cellular Component (CC FAT), and Molecular Function (MF FAT), as well as KEGG pathways [37]. Terms with Benjamini–Hochberg adjusted *p*-value < 0.05 were considered significantly enriched.

### 2.7. Reactome Pathway Enrichment Analysis

Reactome pathway enrichment analysis was performed using the ReactomePA package in R [38]. Human ortholog Entrez Gene IDs were utilized as the input, and separate analyses were conducted for upregulated and downregulated gene sets from each comparison. Pathways with adjusted *p*-value < 0.05 (Benjamini–Hochberg correction) were considered significantly enriched.

### 2.8. Transcription Factor Enrichment Analysis

Transcription factor (TF) enrichment analysis was performed using the Enrichr platform [39,40]. DEGs from each comparison were analyzed against the ChEA 2016 database [41], which contains TF–target gene associations derived from chromatin immunoprecipitation sequencing (ChIP-seq) experiments. Enrichment scores were calculated as −log10 (*p*-value), and results were subsequently visualized as heatmaps using the ComplexHeatmap package in R [42]. TFs showing significant enrichment (*p* < 0.05) across multiple comparisons were specifically highlighted.

### 2.9. Protein–Protein Interaction Network Analysis

Protein–protein interaction (PPI) networks were constructed using the STRING database (version 11.5) [43] via the STRINGdb R package [44]. Human orthologs of DEGs (|fold change| ≥ 1.8, adjusted *p* < 0.05) were mapped to STRING identifiers and interactions with a combined score ≥ 400 (medium confidence) were retained. Network visualization was performed using the ggraph [45] and tidygraph [46] packages with force-directed layouts (Fruchterman–Reingold algorithm). Community detection was performed using the Louvain algorithm [47] to identify putative functional modules within the networks. Node properties including degree centrality, betweenness centrality, and closeness centrality were calculated using the igraph package [48]. Hub genes were defined as nodes with degree values in the top 25th percentile. Network findings are derived from conserved mammalian protein–protein interactions and should therefore be interpreted with caution, as they reflect cross-species regulatory relationships rather than rat-specific interaction networks.

### 2.10. Data Visualization

Volcano plots were generated using ggplot2 [49] to visualize log2 fold changes versus −log10 adjusted *p*-values, with genes colour-coded according to their regulation status. The five most significantly altered genes in each direction were annotated with gene symbols using the ggrepel package [50]. Venn diagrams illustrating overlapping DEGs between comparisons were created using the ggvenn package [51], with shared genes annotated directly on the plots. Heatmaps displaying expression patterns of top-ranked genes were generated using the ComplexHeatmap package [42] with the pheatmap function. Expression values were row-scaled (z-scores), and samples were annotated by treatment group. Adjacent heatmaps were used to display fold-change values for each comparison with custom colour scales. Box-and-whisker plots for ELISA data were generated using ggplot2 with the ggprism theme [52]. Dot plots for GO and pathway enrichment were created using the clusterProfiler [53] and enrichplot [54] packages.

### 2.11. Data Availability

Raw microarray data and processed expression matrices have been deposited in the Gene Expression Omnibus (GEO) repository at the National Center for Biotechnology Information (https://www.ncbi.nlm.nih.gov/geo/) (accessed on 9 November 2025) under accession number GSE317604. All R scripts used for data analysis are available upon request.

## 3. Results

**Figure 1:**
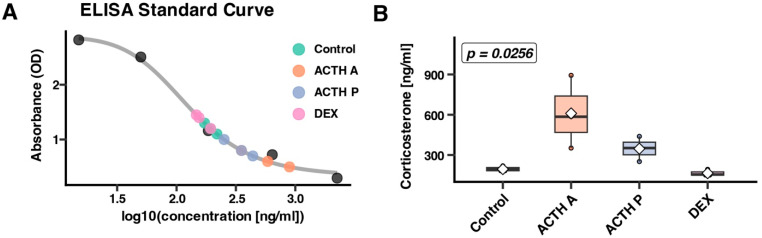
Plasma corticosterone levels in response to ACTH and Dexamethasone. (**A**) ELISA standard curve with individual sample absorbance values. Coloured circles represent experimental groups: Control (green), ACTH A—acute ACTH (orange), ACTH P—prolonged ACTH (blue), and DEX—Dexamethasone (pink). (**B**) Plasma corticosterone concentrations in experimental groups. Box plots show median (line), interquartile range (box), range (whiskers), mean (diamond), and individual values (circles). Kruskal–Wallis test: *p* = 0.0256. The black dots indicate the ELISA standard concentrations used to generate the calibration curve, while the gray line represents the fitted standard curve used to interpolate sample concentrations.

To evaluate the functional steroidogenic response of the adrenal cortex under distinct treatment regimens, we quantified plasma corticosterone concentrations in rats subjected to acute ACTH stimulation (1 h after single injection) reflecting an immediate transcriptional response, prolonged ACTH exposure (12 h after three injections at 0, 12, and 24 h) representing repeated stimulation, Dexamethasone-mediated HPA suppression, or vehicle control (Figure 1). Significant inter-group differences were observed (Kruskal–Wallis test, *p* = 0.026). Acute ACTH is shown to induce the highest corticosterone secretion. Animals receiving a single ACTH injection (ACTH A) and sacrificed 1 h post-administration exhibited markedly elevated plasma corticosterone (610.1 ± 156.8 ng/mL), nearly twofold higher than those observed in control animals (319.9 ± 13.3 ng/mL). Prolonged ACTH exposure is consistent with attenuation of steroidogenic output. Despite receiving three sequential ACTH injections over 36 h, the ACTH P group displayed intermediate corticosterone levels (347.3 ± 54.2 ng/mL) that were significantly lower than those observed in acutely stimulated animals. Dexamethasone treatment effectively suppressed endogenous corticosterone production. Prolonged Dexamethasone administration was associated with reduced plasma corticosterone to 164.8 ± 4.4 ng/mL, representing approximately half of the control values.

### 3.1. Transcriptional Profiling Reveals Treatment-Specific Gene Expression Signatures

**Figure 2:**
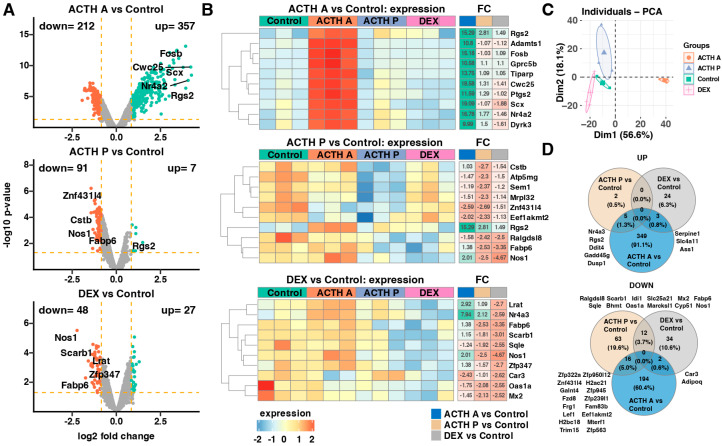
Transcriptomic analysis of rat adrenal glands following ACTH and Dexamethasone treatment. (**A**) Volcano plots illustrating differential gene expression for three pairwise comparisons: acute ACTH (ACTH A vs. Control), prolonged ACTH (ACTH P vs. Control), and Dexamethasone treatment (DEX vs. Control). Genes meeting the significance threshold (adjusted *p*-value < 0.05 and |fold change| ≥ 1.8) are highlighted in green (upregulated) or red (downregulated). Orange dashed lines denote the significance thresholds. The number of significantly downregulated and upregulated genes is indicated in the upper left and right corners of each plot, respectively. Representative top-regulated genes are labelled. (**B**) Heatmaps displaying the expression profiles of the 10 genes with the highest absolute fold-change values across comparisons. Each heatmap shows individual sample expression levels (left panels, colour-coded by z-score), gene symbols, and fold-change (FC) values (right panels). Numerical FC values are presented only for genes and comparisons that meet the predefined significance criteria (adjusted *p*-value < 0.05 and |FC| ≥ 1.8). (**C**) Principal component analysis (PCA) plot based on the most variable genes. Each point represents a single sample, colour-coded according to the experimental group. PC1 and PC2 explain 56.6% and 18.1% of the total variance, respectively. (**D**) Venn diagrams summarizing the overlap of differentially expressed genes between comparisons. The upper diagram corresponds to significantly upregulated genes, while the lower diagram represents downregulated genes. Gene symbols shared between conditions are shown in the corresponding intersecting regions.

To characterize the molecular responses of the adrenal cortex to distinct ACTH regimens and glucocorticoid feedback, we performed genome-wide expression profiling of adrenal tissue using microarray analysis. After quality control filtering (IQR > 0.35) and removal of non-annotated probes, 15,387 transcripts were retained and analyzed for differential expression. Acute ACTH was consistent with the induction of extensive transcriptional activation. Comparison of ACTH A versus Control revealed 569 differentially expressed genes (DEGs; |fold change| ≥ 1.8, adjusted *p* < 0.05), with 357 upregulated and 212 downregulated genes (Figure 2A, upper panel). The most highly induced genes included immediate-early transcription factors (*Fosb*, *Nr4a2*) and stress-responsive genes (*Rgs2*). Heatmap analysis of the top 10 most-changed genes demonstrated robust and selective upregulation specifically in the ACTH A group, with fold changes ranging from 9.99 to 19.09 (Figure 2B, upper panel). In contrast, prolonged ACTH exposure produces a rather moderate transcriptional response. The ACTH P versus Control comparison identified only 98 DEGs (7 upregulated, 91 downregulated), representing a markedly smaller transcriptional footprint than that observed following acute ACTH stimulation (Figure 2A, middle panel). Among the downregulated genes, we observed reduced expression of protease regulators (*Cstb*), mitochondrial proteins (*Atp5mg*, *Mrpl32*), chromatin- and translation-related factors (*Sem1*, *Eef1akmt2*), and lipid-binding or nitrogen-oxide-producing molecules (*Fabp6*, *Nos1*). Fold changes ranged from −2.3 to −2.7 across these genes. Notably, the stress-responsive regulator Rgs2 remained upregulated (fold change +2.81) under both acute and prolonged ACTH stimulation. DEX treatment resulted in the identification of 75 DEGs (27 upregulated, 48 downregulated) compared with control animals (Figure 2A, lower panel). The most strongly suppressed genes were primarily associated with steroid and lipid metabolic pathways, including the cholesterol biosynthesis enzyme *Sqle*, lipid transport and binding proteins (Scarb1, Fabp6), and the retinoid-metabolizing enzyme *Lrat*. In addition, the stress-responsive nuclear receptor Nr4a3 was markedly downregulated. Fold changes ranged from −2.52 to −4.67 across these genes (Figure 2B, lower panel). PCA based on the 1000 most variable genes revealed clear separation of experimental groups along PC1 (56.6% variance) and PC2 (18.1% variance) (Figure 2C). ACTH A samples clustered distinctly from all other groups along PC1, while ACTH P and DEX samples showed intermediate positions between ACTH A and Control, indicating partially overlapping transcriptional states. Venn diagram analysis revealed limited overlap between treatment-induced gene sets (Figure 2D). Most genes upregulated in response to stimulation were unique to acute ACTH exposure (349 genes, 91.1%), with only small subsets shared across ACTH P and DEX conditions. Downregulated genes showed a similar pattern, with acute ACTH accounting for the largest proportion of unique changes (194 genes, 60.4%). In contrast, prolonged ACTH and DEX treatments shared a notable fraction of suppressed genes (12 genes). The overlapping downregulated genes included components of sterol and lipid metabolic pathways (*Sqle*, *Scarb1*, *Fabp6*), retinoid and amino-acid metabolism (*Idi1*), and interferon-stimulated genes (*Oas1a*, *Mx2*). 

### 3.2. Gene Ontology Enrichment Analysis Reveals Treatment-Specific Functional Signatures

**Figure 3:**
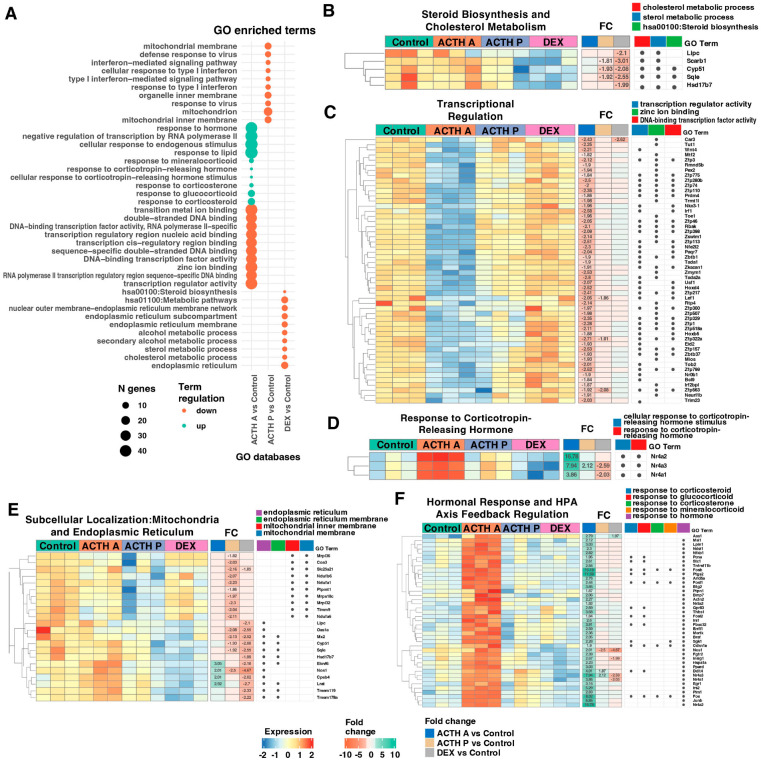
Gene Ontology enrichment analysis of differentially expressed genes in rat adrenal glands following ACTH and Dexamethasone treatment. (**A**) Dot plots summarizing GO enrichment results from three comparisons: acute ACTH (ACTH A vs. Control), prolonged ACTH (ACTH P vs. Control), and Dexamethasone treatment (DEX vs. Control). Differentially expressed genes were annotated and analyzed using the DAVID database. Dot size indicates the number of genes within each GO term; colour represents the direction of regulation (red = downregulation, torques = upregulation). (**B**–**F**) Heatmaps displaying detailed expression profiles of genes associated with enriched GO terms. Each row represents a gene and each column an individual sample. Panel (**B**) shows genes involved in steroid biosynthesis and cholesterol metabolism. Panel (**C**) shows transcriptional regulators. Panel (**D**) focuses on genes responsive to corticotropin-releasing hormone. Panel (**E**) presents genes encoding mitochondrial and endoplasmic reticulum components. Panel (**F**) displays genes related to hormonal response and HPA axis feedback regulation. Colour intensity reflects normalized expression values; fold-change (FC) values are displayed numerically for significant comparisons (adjusted *p*-value < 0.05). Dot annotations indicate GO term classification.

To gain insight into the biological processes modulated by distinct ACTH regimens and glucocorticoid feedback, we performed Gene Ontology (GO) enrichment analysis using the DAVID functional annotation tool. Differentially expressed genes (|fold change| ≥ 1.8, adjusted *p* < 0.05) from each comparison were mapped to human orthologs and analyzed for enrichment in biological processes (BP FAT), cellular components (CC FAT), molecular functions (MF FAT), and KEGG pathways (Benjamini–Hochberg correction, *p* < 0.05). Acute ACTH exposure was associated with the induction of transcriptional programmes linked to stress response and cell signalling. The ACTH A vs. Control comparison revealed a prominent upregulation of genes involved in transcription (42 genes, *p* = 4.65 × 10^−8^), RNA polymerase activity (34 genes, *p* = 4.66 × 10^−8^), and zinc ion binding (43 genes, *p* = 8.84 × 10^−8^) (Figure 3A, upper panel). GO terms related to cellular response to external stimuli were highly enriched, including “response to corticotropin-releasing hormone” (13 genes, *p* = 1.14 × 10^−6^), “response to glucocorticoid” (12 genes, *p* = 1.63 × 10^−6^), and “cellular response to hormone stimulus” (49 genes, *p* = 7.20 × 10^−8^). Collectively, these findings reflect the rapid transcriptional activation characteristic of acute ACTH stimulation. Prolonged ACTH treatment was associated with transcriptional signatures indicative of metabolic adaptation and modulation of mitochondrial function. In contrast to acute stimulation, the ACTH P vs. Control comparison showed predominant downregulation of genes linked to mitochondrial processes, including “mitochondrial inner membrane” (19 genes, *p* = 2.47 × 10^−5^) and “mitochondrial translation” (11 genes, *p* = 1.93 × 10^−5^) (Figure 3A, middle panel). Additionally, genes related to type I interferon response and defence mechanisms were suppressed, including “response to type I interferon” (6 genes, *p* = 2.62 × 10^−6^) and “cellular response to interferon-beta” (5 genes, *p* = 3.81 × 10^−6^). Dexamethasone treatment was associated with the suppression of steroidogenic and metabolic pathways. DEX treatment resulted in marked downregulation of genes involved in cholesterol and steroid metabolism (Figure 3A, lower panel). The most significantly enriched terms included “endoplasmic reticulum” (12 genes, *p* = 5.81 × 10^−6^), “cholesterol metabolic process” (5 genes, *p* = 1.80 × 10^−4^), “sterol metabolic process” (5 genes, *p* = 1.09 × 10^−4^), and “alcohol metabolic process” (6 genes, *p* = 1.64 × 10^−5^). The KEGG pathway “Steroid biosynthesis” (hsa00100) was also significantly suppressed (11 genes, *p* = 4.78 × 10^−7^), consistent with the inhibitory effects of glucocorticoid negative feedback on adrenal steroidogenesis. Additional downregulated terms included “endoplasmic reticulum outer membrane” (8 genes, *p* = 6.00 × 10^−5^) and “endoplasmic reticulum inner membrane” (8 genes, *p* = 3.48 × 10^−5^), reflecting a comprehensive suppression of the steroidogenic machinery. Detailed expression profiling revealed coordinated regulation within functional gene modules. Heatmap analysis of genes within key GO categories demonstrated highly coordinated expression patterns across experimental groups (Figure 3B–F). Steroid biosynthesis and cholesterol metabolism (Figure 3B) were characterized by coordinated downregulation of multiple genes including *Lrat*, *Nr4a3*, *Fabp6*, *Scarb1*, *Sqle*, *Nos1*, *Zfp347*, *Car3*, *Oas1a*, and *Mx2* in DEX-treated animals (fold changes −1.99 to −3.01). Transcriptional regulators and DNA-binding proteins (Figure 3C) including zinc finger proteins (*Zfp113*, *Zfp1*, *Zfp329*, *Zfp280b*, *Zfp322a*, *Zfp563*) exhibited predominant downregulation in response to acute ACTH stimulation. Notably, *Car3*, *Tut1*, *Wnt4*, and *Zfp3* showed suppression in the ACTH A group (fold changes −2.62 to −2.12), while *Zmym1*, *Tada2a*, *Usf1*, and *Hoxd4* demonstrated coordinate downregulation. Prolonged ACTH treatment resulted in more modest changes, with *Lef1*, *Zfp322a*, and *Zfp563* showing downregulation. Genes involved in the corticotropin-releasing hormone response (Figure 3D) displayed marked treatment-specific regulation. Members of the nuclear receptor subfamily 4 showed strong upregulation following acute ACTH stimulation, with *Nr4a2* (fold change 16.78), *Nr4a3* (fold change 7.94), and *Nr4a1* (fold change 3.86) demonstrating robust induction in the ACTH A group. Prolonged ACTH treatment resulted in modest upregulation of *Nr4a3* (fold change 2.12), while Dexamethasone suppressed both *Nr4a3* (fold change −2.59) and *Nr4a1* (fold change −2.03). Subcellular annotation analysis revealed coordinated repression of genes encoding mitochondrial inner membrane components, mitochondrial ribosomal subunits, and ER-resident proteins across treatments (Figure 3E). Prolonged ACTH treatment was associated with broad downregulation of mitochondrial pathways, including *Timm9*, *Slc25a21*, *Mrps18c*, *Mrpl32*, *Ndufa1*, *Ndufa6*, *Mrpl36*; (FC −1.82 to −2.30). In contrast, acute ACTH selectively upregulated signalling regulators (*Nos1*, FC +2.01; *Elovl6*, FC +3.05) without suppressing mitochondrial structural genes. DEX treatment exerted the strongest repression of ER-localized metabolic enzymes, including *Sqle* (FC −2.55), *Elovl6* (FC −2.18), and *Lrat* (FC −2.70), alongside interferon-stimulated genes (*Oas1a*, *Mx2*; FC −2.52 to −2.55). Acute ACTH selectively activated immediate-early and hormone-responsive genes, with limited induction observed under prolonged ACTH exposure or DEX treatment (Figure 3F). The dominant acute transcriptional response comprised classical AP-1 factors (*Fos*, *Fosb*, *Fosl1*, *Fosl2*, *Junb*, *Egr1*; FC 2.4–15.2), stress-response regulators (*Ddit4*, *Cdkn1a*, *Btg2*), and nuclear receptors *Nr4a1–3* and *Ppard* (FC 3.0–16.8). Notably, *Nr4a3* showed divergent regulation-upregulated by ACTH but suppressed by DEX (FC −2.59). Prolonged ACTH treatment produced a transitional transcriptional pattern, characterized by attenuated early-response gene induction and repression of *Nos1* (FC –2.50) and metabolic regulators. DEX elicited a distinct expression profile characterized by repression of inflammatory/antiviral genes (*Nos1*, *Oas1a*, *Mx2*) and ER-localized metabolic enzymes (*Insig1*, *Elovl6*, *Sqle*), with only partial preservation of AP-1 induction. The table presents DEGs identified by microarray analysis (Affymetrix RaGene 1.1 ST Array) in rat adrenal cells is presented in Supplementary Table S1. 

### 3.3. Reactome Pathway Enrichment Analysis

**Figure 4:**
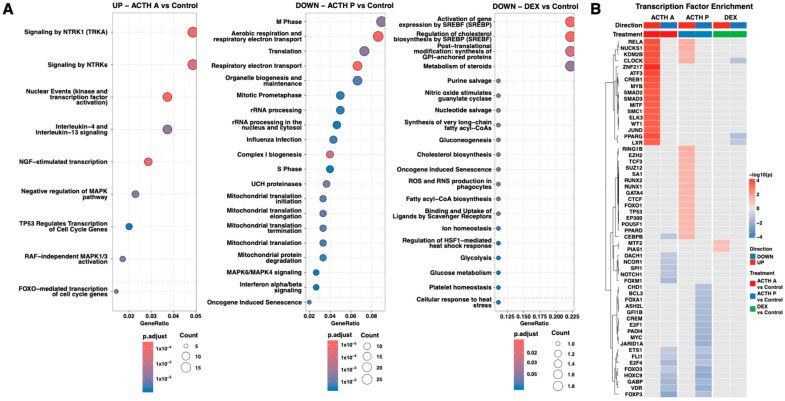
Functional pathway and transcription factor enrichment analysis. (**A**) Reactome pathway enrichment for differentially expressed genes. Dot plots show the top 20 enriched pathways for upregulated genes in ACTH A vs. Control (left), downregulated genes in ACTH P vs. Control (middle), and downregulated genes in DEX vs. Control (right). Dot size: gene count; colour: adjusted *p*-value; dashed line: *p* = 0.05. Enrichment criteria: fold change ≥ 1.8, *p* < 0.05. (**B**) Transcription factor enrichment heatmap (ChEA 2016 database). Top 50 TFs ranked by enrichment signal across comparisons. Colour scale: −log_10_ (*p*-value), positive (red) for upregulated negative (blue) for downregulated gene sets. Column annotations indicate comparison (ACTH A, ACTH P, DEX) and direction (UP/DOWN). n = 3 per group.

To explore coordinated biological responses underlying differential gene expression, we performed Reactome pathway enrichment analysis for all comparisons, separately for up- and downregulated genes (p.adjust < 0.05) (Figure 4A). Acute ACTH treatment resulted in nine significantly enriched pathways, predominantly related to growth factor signalling and transcriptional activation, including nuclear events (kinase and transcription factor activation), NTRK1/NGF-stimulated transcription, negative regulation of MAPK pathway, and FOXO/TP53-mediated transcriptional programmes. These findings are consistent with the rapid activation of immediate-early transcriptional networks associated with stress responsiveness. No pathways reached significance among downregulated genes. In contrast, prolonged ACTH treatment exhibited an inverse enrichment pattern. The upregulated genes yielded no significant pathway enrichment, whereas downregulated genes showed enrichment across pathways converging on aerobic respiration, mitochondrial electron transport, Complex I biogenesis, mitochondrial translation, rRNA processing, and cell cycle regulation. This pattern is consistent with suppression of core mitochondrial and biosynthetic functions and may reflect metabolic down-shifting. Dexamethasone treatment was characterized by coherent biological clusters among downregulated genes centred on SREBF-mediated gene activation and cholesterol biosynthesis regulation, steroid metabolism, post-translational modification. Collectively, these results indicate selective suppression of SREBP-dependent cholesterol and lipid biosynthesis pathways.

### 3.4. Transcription Factor Enrichment Analysis

Transcription factor enrichment analysis revealed distinct regulatory programmes induced by each treatment condition (Figure 4B). Acute ACTH treatment exhibited strong positive enrichment, dominated by TFs associated with immediate-early gene activation and cAMP–PKA signalling. The most upregulated factors included *ZNF217*, *ATF3*, *CREB1*, *KDM2B*, *NUCKS1*, *CLOCK*, *MYB*, *SMAD2/3*, *MITF*, *JUND*, *LXR*, *WT1*, *PPARG*, and *RELA* (−log10(p) 11.48–18.94). Concurrent negative regulation was observed for *FOXP3*, *GABP*, *FOXM1*, *E2F4*, *HOXC9*, *NOTCH1*, *SPI1*, *CEBPB*, *NCOR1*, *DACH1*, *ETS1*, and *FOXO3*, indicating repression of metabolic, proliferative, and immune pathways. Prolonged ACTH treatment displayed a mixed regulatory profile with modest positive enrichment of chromatin regulators and genome stability factors (*RING1B*, *EZH2*, *SUZ12, CTCF*, *SA1*, *KDM2B*, *RUNX1/2*, *TP53*, *POU5F1*, *EP300*, *TCF3*, *FOXO1*, *GATA4*, *CEBPB*, *PPARD*; −log10(p) 1.46–2.37). Strong negative regulation was observed for *HOXC9*, *FOXO3*, *GABP*, *VDR*, *JARID1A*, *MYC*, *FLI1*, *PADI4*, *E2F1*, *CREM*, *GFI1B*, *ASH2L*, *FOXA1*, *FOXP3*, *E2F4*, and *ETS1*, reflecting broad suppression of proliferative, metabolic, and differentiation programmes. DEX treatment induced a selective repressive transcription factor profile distinct from ACTH-mediated responses, which may result from both direct glucocorticoid receptor signalling and reduced ACTH drive. Downregulated TFs included *PPARG, CLOCK*, and *LXR*, while positive enrichment was limited to *MTF2* and *PIAS1*, consistent with canonical GR-mediated transrepression targeting metabolic and circadian regulators.

### 3.5. Protein–Protein Interaction Network Analysis

**Figure 5:**
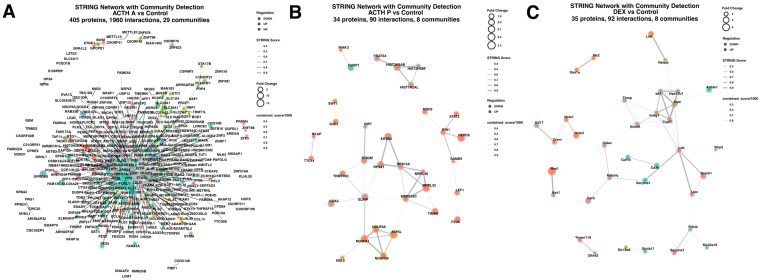
Protein–protein interaction networks reveal treatment-specific molecular signatures. STRING networks with community detection for (**A**) ACTH A vs. Control (405 proteins, 1960 interactions, 29 communities), (**B**) ACTH P vs. Control (34 proteins, 90 interactions, 8 communities), and (**C**) DEX vs. Control (35 proteins, 92 interactions, 8 communities). Nodes: proteins coloured by regulation (green = up, orange = down), size = fold change. Edges: protein interactions, thickness = STRING confidence score. Node borders: community membership (Louvain algorithm). ACTH A induced extensive up-regulation of transcription factors and signalling molecules. ACTH P downregulated mitochondrial and ribosomal proteins. DEX suppressed steroidogenic and lipid metabolism genes. Networks constructed using STRING v11.5 (human database, score ≥ 400), force-directed layout. n = 3 per group, fold change ≥1.8, adjusted *p* < 0.05.

Protein–protein interaction networks were constructed using STRING (v11.5, score ≥ 400) for differentially expressed proteins coding by genes (fold change ≥ 1.8, adjusted *p* < 0.05, n = 3 per group), with community detection performed using the Louvain algorithm (Figure 5). Acute ACTH treatment was associated with the generation of the most extensive interaction network (405 proteins, 1960 interactions, 29 communities), characterized predominantly by up-regulation. Major hubs included transcriptional regulators (FOS, ATF3, EGR1, JUNB, NR4A family, KLF4/6, GADD45A/B/G, CREM, DUSP family, NFE2L2, PIM1), chaperones and proteostasis factors (HSPA1A/B, BAG3, HSPB8, DNAJA4), signalling kinases and metabolic regulators (KRAS, IRS1/2, SGK1, PDK4, FGFR2, BRAF, TXNIP, SOCS2), lipid metabolism enzymes (DGAT1/2, LPIN1, ELOVL6), and cytokine/adhesion proteins (ICAM1, VCAM1, PTGS2, THBS1, PDGFB, ADIPOQ). Downregulated nodes were sparse, consistent with a predominantly activating response. Prolonged ACTH exposure yielded a smaller interaction network (34 proteins, 90 interactions, 8 communities) characterized mainly by downregulation of mitochondrial and ribosomal proteins. Repressed communities included ribosomal subunits (RPS15A, MRPS18C, MRPL32, MRPL36), mitochondrial translation and RNA processing factors (SLIRP, TFB2M, EIF2S2, TRMT10C, COA3), and electron transport chain components (NDUFA1, ATP5L, NDUFA6, NDUFB6). Additional communities contained histone-associated proteins (HIST2H2AB, HIST2H2BF, YEATS4) and signalling/immune regulators (DUSP1, IRAK3, IFIH1, STAT2). DEX treatment generated a network of comparable size (35 proteins, 92 interactions, 8 communities), but with a different composition. Repressed proteins clustered in cholesterol/sterol biosynthesis (SQLE, INSIG1, ELOVL6, CYP51, HSD17B7, IDI1), interferon response (OAS1A, MX2), and lipid transport/metabolism (LDLR, LIPC, SCARB1, FETUB). Additional repressed factors included transcriptional regulators (CREM, NR4A1/3) and metabolic enzymes (CPT1B, CD36, CIDEC, LRAT).

## 4. Discussion

The present study provides a comprehensive transcriptomic analysis of rat adrenal cortex responses to distinct patterns of ACTH stimulation and glucocorticoid feedback. Our results identify three distinct molecular programmes: acute ACTH administration (1 h after single injection, reflecting an immediate transcriptional response) induces widespread transcriptional activation dominated by immediate-early genes; prolonged ACTH exposure (12 h after three injections at 0, 12, and 24 h, representing repeated stimulation) induces metabolic suppression centred on mitochondrial function; and Dexamethasone treatment selectively inhibits cholesterol biosynthesis and uptake pathways, collectively demonstrating the functional plasticity of adrenocortical cells in response to hormonal signals. Our whole-tissue analytical approach captured the integrated transcriptional response of this complex multicellular organ, which was precisely our experimental objective. We sought to characterize the overall molecular programmes governing adrenal responsiveness to ACTH and glucocorticoids at the organ level, reflecting the integrated physiological reality of HPA axis regulation where hormonal signals affect multiple cell types simultaneously and where inter-zonal crosstalk plays important regulatory roles [55]. This strategy enabled the detection of coordinated responses across adrenal zones and cell types, including paracrine signalling between cortical zones, functional interactions between the cortex and medulla, and contributions from supporting cell populations, such as endothelial cells and immune cells that play increasingly recognized roles in adrenal function [56,57]. Collectively, these findings extend previous observations [17,18] and support the concept that temporal patterns of ACTH exposure engage qualitatively different transcriptional programmes, with implications for understanding HPA axis adaptation and glucocorticoid-induced adrenal suppression.

### 4.1. Acute ACTH Stimulation Activates Immediate-Early Gene Networks

Acute ACTH stimulation was associated with the most extensive transcriptional response (569 DEGs, 63% upregulated genes). Among these, genes associated with the canonical cAMP-PKA-CREB signalling cascade were prominently enriched, consistent with the well-established mechanism whereby ACTH binding to the melanocortin 2 receptor (MC2R) stimulates adenylyl cyclase, leading to cAMP accumulation and PKA activation [5,11]. Previous studies using Y1 mouse adrenocortical cells have demonstrated that PKA-dependent signalling accounts for approximately 56% of ACTH-regulated transcripts [17]. Among upregulated genes, *Nr4a* family members (*Nr4a1*, *Nr4a2*, *Nr4a3*; fold changes 3.86-16.78) represented the strongest induction. These orphan nuclear receptors are established immediate-early response genes that are rapidly induced by cAMP-PKA signalling and bind to promoter regions of steroidogenic enzyme genes including *StAR* and *CYP11A1* [12,58,59]. The stress-induced expression of *Nr4a* genes in the adrenal gland has been confirmed in vivo, with marked induction observed following restraint stress in mice [60]. The magnitude of *Nr4a* upregulation in our acute ACTH group (up to 16.78-fold for *Nr4a1*) is consistent with their role as primary transcriptional mediators of ACTH signalling.

Alongside Nr4a receptors, we observed strong induction of *AP-1* transcription factor components, including *Fos*, *Fosb*, *Fosl1*, *Fosl2*, and *Junb* (fold changes up to 15.2). AP-1 family members are also immediate-early genes that respond to both, PKA and MAPK signalling pathways [13,61,62]. Previous studies in rodents have demonstrated that immobilization stress rapidly induces *c-fos* and *junB* expression in the adrenal gland, with peak levels observed within 30-60 min [61]. Our ChEA enrichment analysis suggested *CREB1*, *ATF3* and components of the *AP-1* complex as key regulatory nodes, consistent with the central role of cAMP-responsive element binding protein in mediating ACTH effects [6]. Together, these transcription factors coordinate a broader transcriptional programme that supports sustained steroidogenic output. Furthermore, our Reactome pathway analysis revealed enrichment of *NTRK1/NGF* transcription and *FOXO/TP53* programmes, suggesting that acute ACTH stimulation engages broader cellular stress response and survival mechanisms beyond immediate steroidogenic effects.

The acute ACTH response extended beyond immediate-early genes to include cellular stress response pathways. Heat shock proteins HSPA1A and HSPA1B were among the hub genes in our protein–protein interaction network. HSP70 family members function as molecular chaperones that maintain protein folding capacity during cellular stress [63]. The *Hspa1a* promoter contains functional binding sites for CREB, HSF-1, and NF-kB, enabling coordinated regulation during cellular stress responses [64]. Induction of these chaperons during acute ACTH stimulation likely supports the increased protein synthesis demands associated with rapid upregulation of steroidogenic enzyme expression. Consistently, our Gene Ontology enrichment analysis revealed overrepresentation of stress-response pathways, including “response to corticotropin-releasing hormone”, and “cellular response to hormone stimulus”, suggesting that acute ACTH engages coordinated cellular protective mechanisms alongside activation of steroidogenic pathways. 

### 4.2. Prolonged ACTH Exposure Induces Desensitization Through Mitochondrial Suppression

In contrast to the acute condition, prolonged ACTH administration elicited a markedly different transcriptional response pattern. Despite three ACTH injections administered over 36 h, we observed a reduced transcriptional response (98 DEGs, predominantly downregulated) and intermediate plasma corticosterone levels (347.3 ng/mL) substantially lower than acute ACTH despite continued stimulation. This reduced response indicates functional desensitization phenomenon that has been documented at the level of the HPA axis following prolonged CRH or vasopressin administration [65]. Our data extend this observation by indicating coordinated transcriptional changes that may underline desensitization at the adrenal level. 

Prolonged ACTH treatment was associated with marked suppression of mitochondrial gene expression. Downregulated genes included electron transport chain components (*NDUFA1*, *NDUFA6*, *ATP5MG*), mitochondrial ribosomal proteins (*MRPL32*, *MRPL36*, *MRPS18C*), and mitochondrial translation factors. Our Reactome pathway analysis confirmed significant enrichment of aerobic respiration, Complex I biogenesis, and mitochondrial translation pathways among downregulated genes. This observation is particularly relevant given the central role of mitochondria in adrenal steroidogenesis, in which cholesterol transport to the inner mitochondrial membrane by StAR in the rate-limiting step, and CYP11A1 in the mitochondrial matrix catalyzes the first committed step of steroid biosynthesis [9,11]. Previous studies have shown that mitochondrial dysfunction impairs adrenal steroidogenic capacity [66], suggesting that the mitochondrial suppression we observed may mechanistically contribute to the reduced corticosterone output during prolonged ACTH exposure.

The functional significance of mitochondrial suppression during prolonged ACTH exposure requires consideration of the broader context of HPA axis regulation. Chronic glucocorticoid excess causes multiple adverse effects including metabolic dysfunction, immune suppression and bone loss [67]. By suppressing mitochondrial function, prolonged ACTH exposure may activate an adaptive mechanism that limits steroidogenic capacity even when ACTH stimulation persists, thereby protecting against sustained hypercortisolism. This interpretation is consistent with the intermediate plasma corticosterone level observed, which appear sufficient to maintain physiological homeostasis while remaining substantially lower than the acute peak response. 

Among the limited set of genes upregulated during prolonged ACTH exposure, we identified components of Polycomb Repressive Complex 2 (*EZH2*, *SUZ12*, and *RING1B*), which mediate transcriptional silencing through histone H3 lysine 27 trimethylation (*H3K27me3*) [68]. In the mouse adrenal cortex, *EZH2* has been shown to be essential for proper steroidogenic differentiation, and its ablation results in impaired zona fasciculata development and primary adrenal insufficiency [69]. The upregulation of *PRC2* components observed during prolonged ACTH exposure suggests that epigenetic mechanisms may contribute to transcriptional desensitization. Whether *PRC2* directly targets steroidogenic genes or more broadly reshapes the transcriptional landscape requires further investigation using chromatin immunoprecipitation approaches. 

One gene that remained consistently elevated under both acute and prolonged ACTH stimulation was *Rgs2* (Regulator of G-protein Signalling 2), showing 2.81-fold upregulation in the prolonged group. RGS2 accelerates GTP hydrolysis by Gα subunits, thereby terminating G-protein coupled receptor signalling [70]. In human adrenocortical cells, *RGS2* is induced by angiotensin II and functions as a negative feedback regulator of aldosterone production [71,72]. The sustained elevation of *Rgs2* observed in our study suggests a potential role as an intrinsic brake on MC2R signalling. By accelerating signal termination, Rgs2 may limit the adrenal response to continued ACTH stimulation, explaining the attenuated corticosterone levels we observed. This mechanism represents a direct negative feedback mechanism operating at the level of signal transduction, complementing the metabolic and epigenetic changes described above. 

### 4.3. Dexamethasone Suppresses Adrenal Function Through Inhibition of Cholesterol Metabolism

Dexamethasone treatment was associated with 75 DEGs (64% downregulated) and substantially reduced plasma corticosterone levels (164.8 ng/mL, approximately half of control values). This suggests effective HPA axis suppression through glucocorticoid negative feedback, consistent with the well-established effects of exogenous glucocorticoids on the hypothalamus and pituitary gland [15,16], but also reduced ACTH signalling. Importantly, our findings further suggest that Dexamethasone exerts direct effects on adrenal gene expression, predominantly targeting pathways involved in cholesterol biosynthesis and uptake.

Key enzymes in the cholesterol biosynthesis pathway were significantly downregulated, including squalene epoxidase (*Sqle*, −2.55-fold), isopentenyl-diphosphate delta isomerase (*Idi1*), and lanosterol 14α-demethylase (*Cyp51*). Currently, cholesterol uptake receptors were suppressed, scavenger receptor class B type 1 (*Scarb1*, −2.52-fold) and LDL receptor (*Ldlr*). The concurrent downregulation of both cholesterol uptake receptors *Scarb1* (SR-BI) and *Ldlr* indicates a dual restriction on cholesterol availability. Under physiological conditions, adrenocortical cells preferentially utilize plasma lipoprotein-derived cholesterol for steroid synthesis, with the majority supplied via LDL receptor-mediated uptake and SR-BI-mediated selective uptake from HDL [11,73]. Simultaneous suppression of biosynthesis and uptake pathways may represent a coordinated reduction in cholesterol availability during glucocorticoid excess, effectively limiting the adrenal capacity for steroid production.

Under physiological conditions, adrenocortical cells acquire cholesterol through both de novo synthesis via the sterol regulatory element-binding proteins (SREBPs) pathway and uptake from circulating lipoproteins [11,73]. The coordinate suppression of both pathways therefore represents a comprehensive reduction in cholesterol availability for steroidogenesis. The mechanism underlying this cholesterol pathway suppression involves *SREBP*, which are master transcriptional regulators of cholesterol and fatty acid biosynthesis [74,75]. Glucocorticoids have been shown to inhibit *SREBP2* processing through induction of *Insig2*, which retains *SREBP* in the endoplasmic reticulum and prevents its nuclear translocation and transcriptional activity [76]. Consistently, our Reactome pathway analysis implies significant suppression of SREBF-mediated gene activation among Dexamethasone-downregulated genes, providing direct support for this mechanism. By attenuating SREBP activation, Dexamethasone may limit substrate availability for steroidogenesis; however, establishing causality would require dedicated functional experiments, including targeted manipulation of SREBP activity, direct assessment of cholesterol availability, and steroidogenesis assays.

Moreover, Dexamethasone treatment was associated with the suppression of interferon-stimulated genes (*Oas1a*, *Mx2*), consistent with the well-established anti-inflammatory and immunosuppressive effects on glucocorticoids [77]. Additionally, we observed a downregulation of CLOCK, a core circadian transcriptional regulator. The adrenal cortex harbours an autonomous peripheral clock that gates steroidogenic capacity in response to ACTH [78,79]. Circadian regulation extends across multiple components of the steroidogenic pathway, and disruption of clock gene expression may contribute to altered temporal patterns of glucocorticoid production. Whether glucocorticoid-induced suppression of CLOCK directly contributes to adrenal dysfunction during chronic steroid therapy remains to be determined and warrants further investigation.

### 4.4. Prolonged ACTH and Dexamethasone Converge on Common Suppressive Pathways

Despite the different mechanisms of action, prolonged ACTH and Dexamethasone treatments were both associated with downregulation of a shared set of 12 genes. These genes are predominantly involved in cholesterol metabolism (*Sqle*, *Scarb1*, *Fabp6*, *Idi1*) and interferon responses (*Oas1a*, *Mx2*). This overlap suggests the presence of convergent suppressive regulatory mechanism. During prolonged ACTH exposure, the elevated endogenous corticosterone likely feeds back on the adrenal gland itself, engaging similar glucocorticoid-responsive pathways that are directly activated by exogenous Dexamethasone. This interpretation is supported by the documented expression of glucocorticoid receptors in adrenocortical cells and experimental evidence for direct glucocorticoid effects on adrenal function [15]. 

The regulation of *Nr4a3* across treatment groups illustrates the coordinated nature of adrenal responses. Acute ACTH stimulation induced robust upregulation (+7.94-fold), prolonged ACTH exposure resulted in attenuated but sustained elevation (+2.12-fold), and Dexamethasone treatment produced marked suppression (−2.59-fold). This pattern, the high activation during acute stimulation, partial desensitization during prolonged stimulation, and suppression during glucocorticoid feedback track the functional steroidogenic state of the gland. Given the established roles of *Nr4a* receptors in regulating steroidogenic enzyme expression [12,58], the observed changes in *Nr4a3* expression may contribute to the corresponding alterations in plasma corticosterone levels across treatment groups. 

Our protein–protein interaction network analysis revealed distinct topologies across conditions. The acute ACTH network (405 proteins, 1960 interactions) was predominantly composed of transcriptional regulators, signalling molecules, and chaperones, reflecting the coordinated activation response. The prolonged ACTH network (34 proteins, 90 interactions) consisted primarily of mitochondrial and ribosomal protein clusters, consistent with the metabolic suppression phenotype. The Dexamethasone network (35 proteins, 92 interactions) was centred on cholesterol biosynthesis enzymes and lipid transport proteins, confirming the selective nature of glucocorticoid-mediated suppression. Collectively, these network differences provide a systems-level view of how different regulatory inputs produce distinct molecular states in adrenocortical cells.

### 4.5. Clinical Implications

These findings are relevant for understanding clinical disorders associated with both glucocorticoid excess and deficiency. In Cushing’s disease, where pituitary adenomas produce autonomous ACTH secretion, patients experience chronic ACTH exposure, which closely parallels the prolonged ACTH paradigm investigated in the present study. Our transcriptional analysis indicate that sustained ACTH stimulation is associated with coordinated suppression of mitochondrial gene expression and enrichment of chromatin-modifying pathways, raising the possibility that adaptive transcriptional and epigenetic remodelling may occur in the adrenal cortex during chronic ACTH excess, potentially modulating disease severity. While these adaptive mechanisms are clearly insufficient to prevent the clinical manifestations of hypercortisolism, their identification provides molecular context for understanding how the adrenal cortex responds to prolonged ACTH drive and may inform future strategies aimed at attenuating glucocorticoid overproduction without necessitating surgical intervention.

For patients undergoing long-term glucocorticoid therapy, our results provide molecular insight into the mechanisms underlying iatrogenic adrenal suppression. By integrating Dexamethasone-induced transcriptional changes with established glucocorticoid feedback mechanisms, we demonstrate that adrenal suppression involves direct transcriptional regulation within the adrenal cortex, rather than reflecting hypothalamic–pituitary axis shutdown alone. The SREBP pathway inhibition and cholesterol metabolism suppression we observed suggest that adrenal suppression during glucocorticoid therapy involves direct transcriptional effects on the adrenal cortex, not only hypothalamic-pituitary axis shutdown. These findings have important implications for adrenal recovery after glucocorticoid withdrawal: the restoration of adrenal function may require reversal of cholesterol pathway suppression and potentially epigenetic reprogramming, not merely restoration of ACTH secretion. The observation that adrenal insufficiency can persist for months to years after glucocorticoid discontinuation [16] is consistent with the involvement of stable epigenetic modifications such as PRC2-mediated histone methylation.

The circadian disruption we observed (CLOCK suppression by Dexamethasone) may further contribute to the complexity of adrenal suppression and subsequent recovery. The adrenal peripheral clock regulates temporal patterns of steroidogenic gene expression and responsiveness to ACTH [78,79]. Glucocorticoid-induced disruption of circadian organization could therefore affect both the magnitude and timing of adrenal responses. Whether strategies aimed at preserving circadian function, such as chronotherapy employing time-restricted glucocorticoid administration, might attenuate the severity or duration of adrenal suppression is an empirical question and warrants systematic clinical investigation.

### 4.6. Limitations

Several important limitations of this study should be acknowledged. First, the use of whole adrenal tissue enabled the assessment of integrated organ-level transcriptional responses but precluded zone- or cell-type-specific resolution. Consequently, potential differences among the zona glomerulosa, fasciculata, and reticularis, as well as cell-specific mechanisms, could not be resolved; future spatial transcriptomic or single-cell approaches [80,81] would provide complementary insight. Second, the sample size (n = 3 per group), while appropriate for discovery-based microarray analyses, may limit sensitivity to subtle transcriptional changes; nevertheless, the study was designed to capture dominant transcriptional responses and overarching regulatory programmes, which it effectively achieves. Third, the exclusive inclusion of male rats prevents the assessment of sex-specific differences in HPA axis regulation, which have been reported previously [82]. Fourth, the lack of time-matched sampling between treatment groups represents an important limitation. Acute ACTH responses were assessed at 1 h post-injection, whereas prolonged ACTH and Dexamethasone effects were evaluated 12 h after the final injection, reflecting distinct phases of the transcriptional response. Accordingly, prolonged ACTH findings should be interpreted as representing an early recovery state following repeated stimulation rather than a pure chronic ACTH response; future studies incorporating time-matched sampling designs would more effectively disentangle temporal dynamics from treatment-specific effects. Fifth, transcriptomic profiling does not capture post-translational regulation, protein activity, or metabolic flux, all of which are critical determinants of steroidogenesis; thus, functional validation using mitochondrial and steroidogenic assays is required to substantiate inferred mechanisms. Sixth, although targeted qPCR validation of selected genes could further refine individual fold-change estimates, this was not the primary objective of the study. Instead, our multi-level validation strategy focused on pathway-, network-, and regulator-level analyses, providing a more comprehensive assessment of coordinated biological programmes underlying HPA axis regulation. Seventh, plasma ACTH levels were not measured following Dexamethasone treatment, limiting our ability to distinguish direct glucocorticoid receptor–mediated effects in the adrenal cortex from indirect effects driven by reduced ACTH stimulation; future studies employing in vitro models or ACTH-replacement studies will be required to resolve these contributions. Finally, reliance on human ortholog mapping for pathway and network analyses may introduce species-specific bias; however, parallel analyses using rat-specific annotations support the robustness of our key findings.

## 5. Conclusions

This study demonstrates that acute (1 h after single injection) and prolonged (12 h after three injections at 0, 12, and 24 h) ACTH exposure engage qualitatively different transcriptional programmes in the rat adrenal cortex. Acute ACTH stimulation elicited robust activation of immediate-early genes including *Nr4a* and *AP-1* family members, along with cellular stress response pathways. In contrast, prolonged ACTH exposure was associated with metabolic desensitization characterized by mitochondrial gene suppression, upregulation of Polycomb repressive complexes, and sustained negative feedback through *Rgs2*. Dexamethasone treatment resulted in selective suppression of cholesterol biosynthesis and uptake through inhibition of *SREBP*-mediated transcription. The convergence of prolonged ACTH and Dexamethasone effects on common cholesterol metabolism genes suggests that both endogenous and exogenous glucocorticoid excess engage similar suppressive mechanisms at the adrenal level. These patterns likely reflect a combination of desensitization to repeated stimulation and temporal differences in gene expression kinetics. The identification of mitochondrial suppression as a desensitization mechanism during prolonged ACTH exposure suggests adaptive responses that may limit steroidogenic capacity during chronic stress. Furthermore, characterization of SREBP pathway inhibition as a primary mechanism of glucocorticoid-induced adrenal suppression has implications for understanding and potentially managing adrenal insufficiency in patients receiving long-term glucocorticoid therapy. Future studies incorporating spatial transcriptomics, increased sample sizes, and integration with proteomic and metabolomic data will further refine our understanding of temporal and spatial patterns of adrenal responses to ACTH and glucocorticoid feedback.

## Data Availability

The raw and processed microarray data supporting the conclusions of this article have been submitted to the ArrayExpress under the accession number E-MTAB-16213. Custom R analysis scripts are available from the corresponding author upon reasonable request.

## Supplementary Materials

The following supporting information can be downloaded at: https://www.mdpi.com/article/10.3390/cimb48020135/s1.

## Abbreviations

The following abbreviations are used in this manuscript:

4PL	Four-parameter logistic
ACTH	Adrenocorticotropic hormone
cAMP	Cyclic AMP
ChIP-seq	Chromatin immunoprecipitation sequencing
CRE	cAMP response element
CREB	cAMP response element-binding protein
CRH	Corticotropin-releasing hormone
CYP11A1	Cytochrome P450 side-chain cleavage enzyme
CYP11B1	11β-hydroxylase
CYP21A2	21-hydroxylase
Cyp51	Lanosterol 14α-demethylase
DAVID	Database for Annotation, Visualization, and Integrated Discovery
DEG	Differentially expressed gene
DEX	Dexamethasone
ELISA	Enzyme-linked immunosorbent assay
FC	Fold change
GO	Gene Ontology
HPA	Hypothalamic–pituitary–adrenal
HSD3B	3β-hydroxysteroid dehydrogenase
Idi1	Isopentenyl-diphosphate delta isomerase
IQR	Interquartile range
LDLR	LDL receptor
MAPK	Mitogen-activated protein kinase
MC2R	Melanocortin 2 receptor
OD	Optical density
PCA	Principal component analysis
PKA	Protein kinase A
PPI	Protein–protein interaction
Rgs2	Regulator of G-protein Signalling 2
RIN	RNA integrity number
RMA	Robust Multiarray Average
Scarb1	Scavenger receptor class B type 1
Sqle	Squalene epoxidase
SR-BI	Cholesterol uptake receptors Scarb1
SREBP	Sterol regulatory element-binding protein
StAR	Steroidogenic acute regulatory protein
TF	Transcription factor
